# ART-Treated Patients Exhibit an Adaptive Immune Response against the HFVAC Peptides, a Potential HIV-1 Therapeutic Vaccine (Provir/Latitude45 Study)

**DOI:** 10.3390/v12111256

**Published:** 2020-11-05

**Authors:** Hervé Fleury, Sabrina Caldato, Patricia Recordon-Pinson, Patricia Thebault, Gwenda-Line Guidicelli, Mojgan Hessamfar, Philippe Morlat, Fabrice Bonnet, Jonathan Visentin

**Affiliations:** 1 Pole de Biologie, CHU de Bordeaux, 33076 Bordeaux, France; 2 CNRS UMR 5234, Université de Bordeaux, 33076 Bordeaux, France; patricia.recordon-pinson@u-bordeaux.fr; 3 Service de Médecine Interne et Maladies Infectieuses, Hôpital Saint André, CHU de Bordeaux et Université de Bordeaux, ISPED INSERM U 1219, 33076 Bordeaux, France; sabrina.caldato@chu-bordeaux.fr (S.C.); mojgan.hessamfar@chu-bordeaux.fr (M.H.); philippe.morlat@chu-bordeaux.fr (P.M.); fabrice.bonnet@chu-bordeaux.fr (F.B.); 4 Laboratoire Bordelais de Recherche en Informatique (LaBri), Université de Bordeaux, 33400 Talence, France; Patricia.Thebault@u-bordeaux.fr; 5 Laboratoire d’Immunologie et Immunogénétique, CHU de Bordeaux, 33076 Bordeaux, France; line-gwenda.guidicelli@chu-bordeaux.fr (G.-L.G.); jonathan.visentin@chu-bordeaux.fr (J.V.); 6 CNRS Immuno ConcEpT, Université de Bordeaux, UMR 5164, 33076 Bordeaux, France

**Keywords:** HIV-1, vaccine, HFVAC, CTL epitopes, archived proviral DNA, HLA I alleles, PD-1, ELISPOT

## Abstract

We proposed a new HIV-1 therapeutic vaccine based on conserved cytotoxic T lymphocyte (CTL) epitopes of archived HIV-1 DNA according to their affinity to the dominant HLA-A and -B alleles of the population investigated. Our proposal (Hla Fitted VAC, HFVAC) was composed of 15 peptides originating from the RT, gag and nef parts of proviral DNA. Our aim was to investigate baseline immune reactivity to the vaccine in HIV-1 chronically infected patients at success of antiretroviral therapy (ART) who would be eligible for a therapeutic vaccine. Forty-one patients were tested. Most of them had been infected with HIV-1 subtype B and all had been receiving successful ART for 2 to 20 years. The predominant HLA-A and -B alleles were those of a Caucasian population. ELISPOT was carried out using the HFVAC peptides. In 22 patients, the PD-1 marker was investigated on CD4+ and CD8+ T cells by flow cytometry in order to evaluate global T cell exhaustion. ELISPOT positivity was 65% overall and 69% in patients exhibiting at least one HLA allele fitting with HFVAC. The percentages of CD4+ and CD8+ T cells expressing PD-1 were high (median values 23.70 and 32.60, respectively), but did not seem to be associated with an impairment of the immune response investigated in vitro. In conclusion, reactivity to HFVAC was high in this ART-treated population with dominant HLA alleles, despite potential cellular exhaustion associated with the PD-1 marker.

## 1. Introduction

Among strategies for a viral cure in patients at success of antiretroviral therapy (ART), HIV-1 therapeutic vaccines have been explored since the initial study of Jonas Salk [1]. With greater understanding of the pathophysiology of the HIV-1 disease, the most promising option has been the stimulation of cytotoxic activity associated with CD8+ cytotoxic T lymphocytes (CTL) using different vaccine constructs expressing CTL epitopes (DNA, recombinant viruses based on MVA, VSV, Ad5, ALVAC canarypox) [2]; other vaccine trials have been designed that were based on peptides such as Vacc-4 [3,4]. However, globally, the results of these approaches have been somewhat disappointing.

The French national agency on AIDS research (ANRS) decided early on to focus on lipopeptides in which peptides are linked to a palmitoyl motif (Lipo5) [5], the lipopeptides being able to be presented to the effective cell through interaction with an HLA allele and the T Cell Receptor (TCR). Our project Provir/Latitude45 is based on this lipopeptide strategy, but our concern was the choice of CTL epitopes, taking into account that viral replication at treatment interruption resumes from archived DNA (while epitopes are generally selected on circulating or reference viruses) and that we must also consider fully the genetic background of the patients and/or the population [6]. We have proposed [7] a multi-epitopic vaccine designed from archived HIV-1 DNA of ART-treated patients and composed of 15 highly conserved peptides with affinity to HLA I alleles A and B, widely expressed in the population investigated (Hla Fitted VACcine: HFVAC). In the present study, we investigated the baseline reactivity to HFVAC of patients at success of ART who would be eligible for a vaccine trial. Enzyme-linked immunosorbent spot assay (ELISPOT) was used, since it is known as a powerful tool to investigate T cell responses [8].

## 2. Materials and Methods

### 2.1. Patients

Forty-one patients were recruited in the outpatient unit of the Internal Medicine and Infectious Diseases Department of Saint André University Hospital in Bordeaux between January 2019 and January 2020. All were HIV-1 chronically infected individuals under successful ART. They had been recruited in a previous phase of the Provir/Latitude45 project, and data on HLA alleles and archived proviral DNA HIV-1 sequences were available (RT and Prot for all patients, Gag and Nef for some of them). Therefore, we had the identity of the viral subtypes; briefly, HIV-1 subtype B was dominant, followed by unidentified strains, CRF02_AG, unknown recombinant forms (URFs), 1 CRF22_01A1, 1 D, 1 F1 and 1 BC.

The data on HLA alleles were blinded and the patients were recruited sequentially with no selection based on genetic HLA background. All subjects gave their informed consent for inclusion before they participated in the study and the samples were anonymized. The study was conducted in accordance with the Declaration of Helsinki and the protocol was approved by the Ethics Committee of Sud Ouest and Outremer (DC 2012/48).

### 2.2. ELISPOT and Flow Cytometry

Blood was taken in heparin tubes and used for ELISPOT and flow cytometry. ELISPOT was used to measure the numbers of IFN-γ-producing PBMCs directed against HIV-1 epitopes and expressed as spot-forming units (SFUs). It was performed according to the Human IFN-γ single-color enzymatic ELISPOT protocol of C.T.L. Europe (Bonn, Germany) on microplates with 200,000 fresh PBMCs/well using pool A (15 HIV-1 peptides of HFVAC) and pools B1, B2 and B3 consisting of subgroups of pool A. The peptides and their affinity to HLA alleles A or/and B are presented in Table 1; the theorical MHC IC_50_ are given using the IEDB analysis resource. Phyto-hemagglutinin (PHA, positive control) and a peptide pool (CEF) consisting of 23 MHC class-restricted viral peptides from human CMV, EBV and influenza virus were used as positive controls. The negative controls were RPMI +/− 0.4% DMSO and MOG (a mixture of 29 peptides of myelin oligodendrocyte glycoprotein). The number of SFUs was measured in a C.T.L. automated ImmunoSpot analyzer; the positivity threshold for each peptide pool or antigen was set at 30 SFUs/10^6^ PBMCs. At least 85% of cells responding with this methodology are CD8+ T cells, but all studies were conducted with unfractionated PBMCs.

#### Flow Cytometry

All patients were routinely followed for their T cell populations using CD45/CD3/CD4/CD8 staining and determination of their absolute counts (BD Multitest CD3/CD8/CD45/CD4 used with BD Trucount Tubes, acquired on a BD FACS Lyric, BD Biosciences). The last 22 included patients were also studied for PD-1 expression by CD4+ and CD8+ T cells acquired on a Fortessa cytometer (BD Biosciences, Franklin Lakes, NJ, USA).

### 2.3. Statistical Analysis

Welch’s t-test was used to determine the *p*-value for comparing the means of CD4+ PD-1+ and CD8+PD-1+ T cells (after checking the normal distributions) when analyzing whether there was reactivity to HFVAC peptides.

## 3. Results

### 3.1. Patient Characteristics, Lymphocyte Immunophenotyping and PD-1 Expression

The patients had been treated by ART for 2 to 20 years at entry to the study. Their viral load was below 50 copies/mL the day of ELISPOT assay, with the exception of SA0118 (1590 copies, but <50 copies/mL three weeks later) and SA0151 (94 copies/mL). The results of lymphocyte immunophenotyping and PD-1 expression are presented in Table 2; the median values for CD4+ and CD8+ T cells were 548 and 541/µL, respectively, while PD-1 marker was expressed by 23.70% and 32.90% of CD4+ and CD8+ T cells, respectively. The nadir of CD4+ T cells ranged from 3 to 663/µL (median: 232/µL).

Therefore, the population studied was composed of HIV-1 chronically infected patients under successful ART. About one third of CD8+ T cells in these patients were expressing the PD-1 marker. We then aimed at evaluating the reactivity of these T cells to HFVAC by ELISPOT.

### 3.2. ELISPOT Reactivity According to HLA Alleles, Archived Viral Sequences and PD-1 Expression

ELISPOT data and HLA alleles are presented in Table 3.

**Figure 1:**
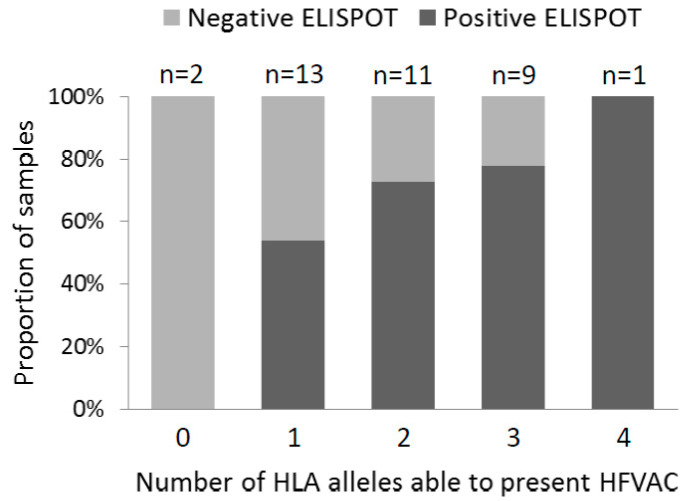
ELISPOT reactivity to HFVAC peptides according to the number of HLA-A and -B alleles fitting with their presentation.

Regarding ELISPOT reactivity to HFVAC peptides, four samples were undetermined (thereafter excluded from the analysis), 13 were negative (35%) and 24 were positive (65%). In the event of positivity in pool A, pool B1 was more frequently reactive, therefore allowing to hypothesize that it contained immune-dominant peptides. Regarding HLA alleles for presentation of the peptides, all patients but two (SA0110 and SA0127) exhibited at least one allele compatible with presentation of an HFVAC peptide; for these patients expressing fitting alleles, the percentage of reactivity was 69%. The number of HLA-A and -B alleles fitting HFVAC was 1 for 13 patients, 2 for 11 patients, 3 for 9 patients and 4 for one patient (homozygous alleles were counted two times; one patient had no HLA-B typing). Figure 1 shows that the proportion of positive patients increased with the number of HLA alleles allowing the presentation of the HFVAC peptides.

**Figure 2:**
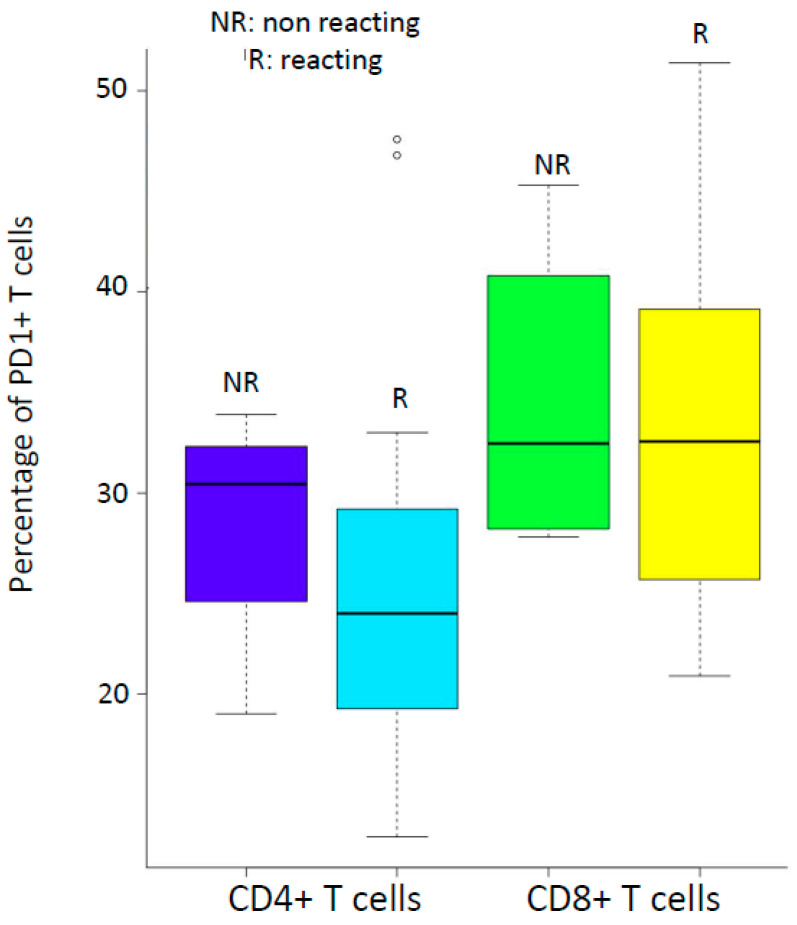
Reactivity to HFVAC peptides by ELISPOT according to the percentages of CD4+ and CD8+ T cells expressing PD-1.

Twenty-two patients were investigated for PD-1 protein associated with CD4+ and CD8+ T cells (Table 2). We then compared the percentages of CD4+PD-1+ in ELISPOT-reactive and nonreactive patients; the same comparison was made for CD8+PD-1+ cells (Figure 2). There were no significant differences in these percentages between reactive and nonreactive patients (CD4+T cells: *t* = 0.65624, df = 7.2029, *p*-value = 0.5321 Welch Two Sample *t*-test; CD8+T cells: *t* = 019264, df = 5.0683, *p*-value = 0.8547 Welch Two Sample *t*-test).

Therefore, a high proportion of the population studied was reactive to HFVAC peptides. Of note, patients 111, 116, 117, 119 and 128 showed negative reactivity which was not fully explained by mutations in the archived epitopes or global PD-1 expression on T lymphocytes (data not shown).

## 4. Discussion

The aim of the study was to establish a baseline value of reactivity to the peptides of HFVAC in a population of HIV-1 chronically infected patients on successful ART. The percentage of reactivity was high overall and higher in patients whose HLA alleles belonged to those eligible for the proposed vaccine. This was consistent with reactivity to peptides according to the genetic background of the patients, as hypothesized in the Provir/Latitude45 project. These baseline ELISPOT data should be compared with those obtained in major HIV therapeutic vaccine trials. In the HTI-TriMix trial, where HIV peptides are not directly injected but generated by corresponding mRNAs [9,10], 31% of the patients were reactive to the so-called IN peptides before initiation of the vaccine program. The percentage of patients included in Vacc-4X [3,4] and who were reactive at baseline was not mentioned in the seminal study [3]; the article by Rockstroh et al. [4] describes a reboost study including patients who had already received Vacc-4X five years before; at week 4 of the reboost vaccine trial, less than 10% of the patients exhibited ELISPOT reactivity to Vacc-4X peptides, and this proportion reached 60% at week 28. Compared to these baseline evaluated ELISPOT values, the Provir/Latitude45 results seem encouraging, particularly if we consider that the immune system of individuals with higher baseline immune responses while still on cART may be more responsive to immune stimulation by vaccination than those who had lower baseline immune responses [11]. As a different approach, Mothe et al. [12], using recombinant viruses (adenovirus prime-poxvirus MVA boost) expressing a conserved part of the HIV-1 proteome were able to propose the concept that subdominant epitopes in a natural infection can induce strong responses in patients on antiretroviral treatment and efficiently refocus HIV-1-specific T-cells to the protective epitopes delivered by the vaccine; it must be noted that the patients of this latter study were close to HIV-1 primary infection, while our first objective was to address chronically infected patients on ART.

We analyzed the percentages of CD4+ and CD8+ T cells expressing the PD-1 marker, since the latter has been associated with T cell exhaustion in HIV-infected patients, as previously demonstrated in cancer patients [13,14]. Although our analysis was limited to 22 patients, the percentages in our HIV-positive patients were much higher than in HIV-negative ones [15] (23.70% versus 6.2% for CD4+T cells and 32.90% versus 16.08% for CD8+T cells). However, there was still an ELISPOT response to the peptides of HFVAC in patients whose PD-1 expression by T cells exhibited high values. We must underline that we were unable to perform ELISPOT experiments on sorted PD1+ and PD- T CD8+ cells and that we have not investigated other checkpoint markers such as CTLA-4, as an example [13].

Finally, we identified patients without any response to HFVAC peptides. The TCR repertoire for ligation of the peptides associated with HLA alleles is clonal, so one hypothesis is the loss of clones at the moment of primary infection or during the period of chronic productive infection before initiation of ART [16]. Four patients exhibited a very low CD4+ T lymphocytes nadir (ranging from 3 to 14/uL) that could be associated with a profound loss of the TCR repertoire among CD8+ T cells; nevertheless, only one of them (SA0119) was negative by ELISPOT while the three other ones (SA0122, SA0131 and SA0146) were reactive.

In conclusion, this study demonstrated that HIV-1 infected patients on ART who have a genetic background similar to that described in our previous Provir/Latitude45 studies exhibit cellular immune reactivity to the peptides of HFVAC. This paves the way for in vivo studies evaluating vaccine potential of HFVAC in animal models. The next step will investigate the ability of the 15 lipopeptides of HFVAC supplemented with an adjuvant to induce a CD8+T cell response in HIS mice.

## Figures and Tables

**Table 1:** HIV-1 peptides of Hla Fitted VACcine (HFVAC) and their presenting HLA alleles. Pool A contained all peptides while pools B1, B2 and B3 contained subgroups of pool A. The theorical MHC IC_50_ is noted in the brackets. All MHC IC_50_ theorical values were <500 nM, with the exception of Nef(134-143)/HLA-A*24:02; this couple peptide/allele has been selected because it is considered in the CTL/CD8+ epitope table of the HIV molecular immunology database (hiv.lan.gov).

Pool	Epitope Identification	Class I Allele(s) with High Affinity to Epitope
A	15 peptides	
B1	RT (181–189)	HLA-A*02:01(11.5 nM)
	RT (158–166)	HLA-A*03:01(12.8 nM), A*11:01(8.3 nM), A*:68:01(176.2 nM)
	RT (110–118)	HLA-A*68:02(20.2 nM)
	RT (163–171)	HLA-B*15:01(24.8 nM)
	Gag (433–440)	HLA-A*02:01(12.5 nM)
B2	RT (227–234)	HLA-A*02:01(17.7 nM)
	RT (73–82)	HLA-A*03:01(20.3 nM), A*11:01(40.8 nM)
	RT (156–164)	HLA-B*07:02(9 nM), B*35:01(43.5 nM)
	Gag (362–370)	HLA-A*02:01(5.9 nM)
	Nef (134–143)	HLA-A*24:02(558.3 nM), A*29:02(144.8 nM)
B3	RT (232–241)	HLA-A*02:01(43 nM)
	RT (240–249)	HLA-A*11:01(17.1 nM), A*68:01(75.9 nM)
	RT (18–26)	HLA-B*08:01(41.5 nM)
	RT (107–115)	HLA-A*29:02(90.2), HLA-B*35:01(21.8 nM)
	Gag (148–156)	HLA-B*07:02(37.3 nM)

**Table 2:** Median values, 1st and 3rd quartiles for total lymphocyte count, T CD4+, T CD8+, T CD4+ PD-1+ and T CD8+ PD-1+ cells. Total lymphocytes, T CD4+ and T CD8+ cell counts were obtained for 41 patients, while PD-1 expression was investigated in 22 samples.

	Median	Q1	Q3
Lymphocytes	1685/µL	1314/µL	2090/µL
CD4+	548/µL	460/µL	742/µL
CD8+	541/µL	331/µL	848/µL
CD4+/CD8+	1	0.8	1.7
CD4+ PD-1+ among CD4+ T cells	23.70%	19.70%	29.80%
CD8+ PD-1+ among CD8+ T cells	32.90%	28.33%	38.98%

**Table 3:** ELISPOT results according to HLA alleles expressed by the patients. The table shows quantitative data of ELISPOT assay in SFUs/10^6^ PBMC according to the peptide pool used: A, pool of 15 peptides; B1, B2 and B3 were subpools, as described in Table 1. The positivity threshold was set at 30 SFUs/10^6^ PBMC. Underlined and bolded alleles are those showing high affinity for one or several peptides of pool A. ND: not determined (negative control MOG showed more than 30 SFUs/10^6^ PBMC). HLA-B typing of patient SA0122 was not available.

Patient ID	ELISPOT Results (SFUs/10 ^6^ PBMC)				ELISPOT Results	Peptide Presentation	HLA-Typing
	Pool A	Pool B1	Pool B2	Pool B3			
SA0107	<30	<30	<30	<30	Negative	Yes	A*29:02,*68:01; B*58:02,*81:01
SA0108	**460**	<30	**380**	<30	**Positive**	Yes	A*02:01,*24:02, B*07:02,
SA0109	**60**	<30	**65**	<30	**Positive**	Yes	A*03:01,*23:01; B*07:02,*58:01
SA0110	<30	<30	<30	<30	Negative	No	A*02:05,*26:01; B*49:01,*55:01
SA0111	<30	<30	<30	<30	Negative	Yes	A*03:01,*26:01; B*38:01,*40:01
SA0116	<30	<30	<30	<30	Negative	Yes	A*02:01,*26:01; B*27:05,*39:01
SA0117	<30	<30	<30	<30	Negative	Yes	A*24:02,*29:02; B*15:01,*51:01
SA0118	<30	<30	<30	<30	Negative	Yes	A*02:01,*68:02; B*53:01,*58:01
SA0119	<30	<30	<30	<30	Negative	Yes	A*03:01,*68:01; B*58:02,-
SA0120	<30	<30	<30	<30	Negative	Yes	A*02:05,*11:01; B*35:03,*50:01
A0121	<30	<30	<30	<30	Negative	Yes	A*01:01,*26:01; B*08:01,*14:01
SA0122	**55**	<30	<30	<30	**Positive**	Yes	A*02:01,*24:02
SA0123	<30	<30	<30	<30	Negative	Yes	A*24:02,*32:01; B*13:02,*39:01
SA0124	<30	**41**	<30	<30	**Positive**	Yes	A*02:01,*03:01; B*07:02,*39:06
SA0125	ND	ND	ND	ND	ND	Yes	A*03:01,*03:02; B*18:01,*49:01
SA0126	<30	**130**	**100**	**65**	**Positive**	Yes	A*11:01,*24:02; B*08:01,*27:05
SA0127	< 30	<30	<30	<30	Negative	No	A*23:01,*34:02; B*15:03,*58:01
SA0128	< 30	<30	<30	<30	Negative	Yes	A*29:01,*68:02; B*49:01,*53:01
SA0129	< 30	<30	<30	<30	Negative	Yes	A*02:01; B*14:01,*15:01
SA0130	**310**	**110**	<30	<30	**Positive**	Yes	A*03:01,*24:02; B*08:01,*40:01
SA0131	**455**	**115**	<30	<30	**Positive**	Yes	A*02:01,*68:01; B*15:17,*51:01
SA0132	**30**	**440**	<30	<30	**Positive**	Yes	A*11:01,*29:02; B*40:01,*44:02
SA0133	**175**	**285**	<30	<30	**Positive**	Yes	A*11:01,-; B*35:01,*55:01
SA0134	**80**	**260**	<30	<30	**Positive**	Yes	A*01:01,*29:02; B*44:03,*57:01
SA0135	ND	ND	ND	ND	ND	Yes	A*01:01,*02:02; B*07:02,*18:01
SA0136	ND	ND	ND	ND	ND	Yes	A*03:01,*25:01; B*08:01,*35:01
SA0137	**115**	**105**	<30	<30	**Positive**	Yes	A*30:02,*68:02; B*15:17,*18:01
SA0138	**555**	**200**	<30	<30	**Positive**	Yes	A*02:01,*30:02; B*41:01,*44:02
SA0139	**215**	**170**	**50**	**30**	**Positive**	Yes	A*03:01,*30:01; B*07:02,*53:01
SA0140	**1025**	**475**	<30	<30	**Positive**	Yes	A*68:01,*74:03; B*51:01,
SA0141	**935**	**375**	<30	<30	**Positive**	Yes	A*02:01,*30:01; B*35:01,*51:01
SA0142	**295**	**110**	<30	<30	**Positive**	Yes	A*03:01,*24:02; B*07:02,*56:01
SA0143	**800**	**275**	<30	<30	**Positive**	Yes	A*24:02,*29:02; B*07:02,*13:02
SA0144	**400**	**365**	<30	<30	**Positive**	Yes	A*01:01,*30:02; B*08:01,*18:01
SA0145	**810**	**640**	<30	<30	**Positive**	Yes	A*02:01,*68:02; B*35:03,*53:01
SA0146	**170**	**70**	<30	<30	**Positive**	Yes	A*03:01,*30:02; B*07:02,*15:10
SA0147	ND	ND	ND	ND	ND	Yes	A*68:02,*74:01; B*44:03,*53:01
SA0148	**1160**	**510**	<30	<30	**Positive**	Yes	A*24:02,*68:02; B*35:02,*50:01
SA0149	**365**	**110**	<30	<30	**Positive**	Yes	A*02:01,*74:01; B*15:10,*53:01
SA0150	**1695**	**1655**	**30**	**90**	**Positive**	Yes	A*03:01,*30:01; B*13:02,*27:05
SA0151	**550**	**445**	<30	<30	**Positive**	Yes	A*01:01,*03:01; B*07:02,*08:01

## References

[1] Salk J., Bretscher P.A., Salk P.L., Clerici M., Shearer G.M. (1993). A strategy for prophylactic vaccination against HIV. Science.

[2] Fleury H., Tumiotto C., Bellecave P., Recordon-Pinson P. (2018). Therapeutic Vaccine Against HIV, Viral Variability, Cytotoxic T Lymphocyte Epitopes, and Genetics of Patients. AIDS Res. Hum. Retrovir..

[3] Pollard R.B., Rockstroh J.K., Pantaleo G., Asmuth D.M., Peters B., Lazzarin A., Garcia F., Ellefsen K., Podzamczer D., Van Lunzen J. (2014). Safety and efficacy of the peptide-based therapeutic vaccine for HIV-1, Vacc-4x: A phase 2 randomised, double-blind, placebo-controlled trial. Lancet Infect. Dis..

[4] Rockstroh J.K., Asmuth D., Pantaleo P., Clotet C., Podzamczer D., Van Lunzen J., Arastéh K., Mitsuyasu R., Peters B., Silvia N. (2019). Re-boost immunizations with the peptide-based therapeutic HIV vaccine, Vacc-4x, restores geometric mean viral load set-point during treatment interruption. PLoS ONE.

[5] Gahéry-Ségard H., Pialoux G., Charmeteau B., Sermet S., Poncelet H., Raux M., Tartar A., Lévy J.P., Gras-Masse H., Guillet J.G. (2000). Multiepitopic B- and T-cell responses induced in humans by a human immunodeficiency virus type 1 lipopeptide vaccine. J. Virol..

[6] Deng K., Pertea M., Rongvaux A., Wang L., Durand C.M., Ghiaur G., Lai J., McHugh H.L., Hao H., Zhang H. (2015). Broad CTL response is required to clear latent HIV-1 due to dominance of escape mutations. Nature.

[7] Tumiotto C., Alves B.M., Recordon-Pinson P., Jourdain M., Bellecave P., Guidicelli G.L., Visentin J., Bonnet F., Hessamfar M., Neau D. (2019). Provir/Latitude 45 study: A step towards a multi-epitopic CTL vaccine designed on archived HIV-1 DNA and according to dominant HLA I alleles. PLoS ONE.

[8] Russell N.D., Hudgens M.G., Ha R., Havenar-Daughton C., McElrath M.J. (2003). Moving to human immunodeficiency virus type 1 vaccine efficacy trials: Defining T cell responses as potential correlates of immunity. J. Infect. Dis..

[9] Leal L., Guardo A.C., Morón-López S., Salgado M., Mothe M., Heirman C., Pannus P., Vanham G., van den Ham H.J., Gruters R. (2018). Phase I clinical trial of an intranodally administered mRNA-based therapeutic vaccine against HIV-1 infection. AIDS.

[10] De Jong W., Leal L., Buyze J., Pannus P., Guardo A., Salgado M., Mothe B., Molto J., Moron-Lopez S., Galvez C. (2019). Therapeutic Vaccine in Chronically HIV-1-Infected Patients: A Randomized, Double-Blind, Placebo-Controlled Phase IIa Trial with HTI-TriMix. Vaccines.

[11] Huang Y., Pantaleo G., Tapia G., Sanchez B., Zhang L., Trondsen M., Hovden A.-O., Pollard R., Rockstroh J., Ökvist M. (2017). Cell-Mediated Immune Predictors of Vaccine Effect on Viral Load and CD4 Count in a Phase 2 Therapeutic HIV-1 Vaccine Clinical Trial. EBioMedicine.

[12] Mothe B., Manzardo C., Sanchez-Bernabeu A., Coll P., Morón-López S., Puertas M.C., Rosas-Umbert M., Cobarsi P., Escrig R., Jose M. (2019). Therapeutic Vaccination Refocuses T-cell Responses Towards Conserved Regions of HIV-1 in Early Treated Individuals (BCN 01 study). EClinicalMedicine.

[13] Mylvaganam G., Yanez A.G., Maus M., Walker B.D. (2019). Toward T Cell-Mediated Control or Elimination of HIV Reservoirs: Lessons From Cancer Immunology. Front. Immunol..

[14] Day C.L., Kaufmann D.E., Kiepiela P., Brown J.A., Moodley E.S., Reddy S., Mackey E.W., Miller J.D., Leslie A.J., DePierres C. (2006). PD-1 expression on HIV-specific T cells is associated with T-cell exhaustion and disease progression. Nature.

[15] Booiman T., Wit F.W., Girigorie A.F., Maurer M., De Francesco D., Sabin C.A., Harskamp A.M., Prins M., Franceschi C., Deeks S.G. (2017). Terminal differentiation of T cells is strongly associated with CMV infection and increased in HIV-positive individuals on ART and lifestyle matched controls. PLoS ONE.

[16] Meyer-Olson D., Brady K.W., Bartman M.T., O’Sullivan K.M., Simons B.C., Conrad J.A., Duncan C.B., Lorey S., Siddique A., Draenert R. (2006). Fluctuations of functionally distinct CD8+ T-cell clonotypes demonstrate flexibility of the HIV-specific TCR repertoire. Blood.

